# Proactive psychosocial follow-up of youth exposed to a terrorist attack: longitudinal study linking interviews and register-based data

**DOI:** 10.1192/bjo.2024.838

**Published:** 2025-03-12

**Authors:** Lise Eilin Stene, Kristin Alve Glad, Synne Øien Stensland, Lisa Govasli Nilsen, Grete Dyb

**Affiliations:** Norwegian Centre for Violence and Traumatic Stress Studies, NKVTS, Oslo, Norway

**Keywords:** Psychosocial interventions, trauma- and stressor-related disorders, primary care, mental health services, patients

## Abstract

**Background:**

Knowledge on efficient approaches to the provision of post-disaster psychosocial care is urgently needed. To prevent unmet healthcare needs, proactive follow-up by municipal contact persons was recommended for survivors of the Utøya youth camp attack in Norway.

**Aims:**

To examine characteristics of the survivors by whether or not they had a contact person in the early (0–5 months), intermediary (5–15 months) and long-term (20–32 months) aftermath of the attack, and to describe the survivors’ experiences with the contact person.

**Method:**

We analysed data from three waves of interviews with survivors conducted 4–5, 14–15 and 30–32 months after the attack, as well as register-based data on the use of mental health services from 3 years before until 3 years after the attack.

**Results:**

Survivors with a contact person early post-attack were less likely to receive care from mental health services concurrently or to have anxiety/depression symptoms subsequently compared with survivors without a contact person in the same period. Survivors with a contact person in the intermediary aftermath were more satisfied with the overall help they received, but also more likely to have long-term anxiety/depression symptoms. Survivors with a contact person in the long term were more likely to be financially disadvantaged. Approximately half of the survivors with a contact person found this highly or very highly useful, whereas one-third found it of little use or not at all useful.

**Conclusions:**

The proactive outreach reached survivors across sociodemographic characteristics during the recommended first year of follow-up, which could be conducive to prevention of unmet healthcare needs. Still, there was considerable variation in the perceived usefulness and duration of the follow-up.

Both man-made and natural disasters represent major threats to our societies and public health, affecting hundreds of millions people every year.^1^ Exposed individuals are at risk of developing a range of mental and physical health problems.^2–5^ A major concern is that unmet healthcare needs are repeatedly observed in the wake of disasters.^6–8^ Moreover, there are substantial differences in psychosocial care responses to disasters across countries.^9^ Despite some divergence, international guidelines for post-disaster psychosocial care commonly recommend support of natural recovery, identification of those with significant difficulties, and provision of access to specialised mental health services (MHS) and other support measures when required.^10–15^ This is in line with stepped-care approaches to providing and monitoring care and treatment for common mental disorders in general.^16^ The United Nations’ Inter-Agency Standing Committee's guidelines for mental health and psychosocial support outline four steps, beginning with ‘basic services and security’, followed by ‘community and family supports’ and ‘focused, non-specialised supports’ and, finally, ‘specialised services’, when the earlier steps are not sufficient.^12^ Proactive outreach to those affected has also been recommended to help connect survivors with services and prevent unmet healthcare needs.^17^ Still, there is a lack of knowledge on the most efficient means of organising and providing post-disaster psychosocial care. Current guidelines are largely founded on a consensus of expert opinions. Consequently, there is an urgent need for research on the provision of psychosocial care in the challenging and unpredictable circumstances of disasters.

In this study, we aimed to gain knowledge that could help improve our practices for providing and strengthening access to post-disaster psychosocial care by investigating a proactive, primary-care-based model of follow-up of youth exposed to a terrorist attack. In the proactive model under study, which is described more comprehensively below, it was recommended that the survivors were followed up by a designated contacted person from their local municipality and offered at least three screening assessments of their health and functioning in the first year after the attack.^18^ Previous research has shown that 84% of the survivors reported contact with a designated contact person in the first 5 months and 55% in the period 5–15 months post-attack. Still, it is unknown whether there were differences between the survivors who had a contact person and those who did not.^19,20^ Hence, our objectives were to examine the sociodemographic characteristics of the survivors, their symptom levels and their use of specialised MHS by whether or not they had contact with a designated contact person in the early (0–5 months), intermediary (5–15 months) and long-term (20–32 months) aftermath of the attack. Furthermore, we aimed to explore the survivors’ experiences with the contact person (e.g. perceived accessibility and usefulness).

## Method

### Setting

**Fig. 1:**
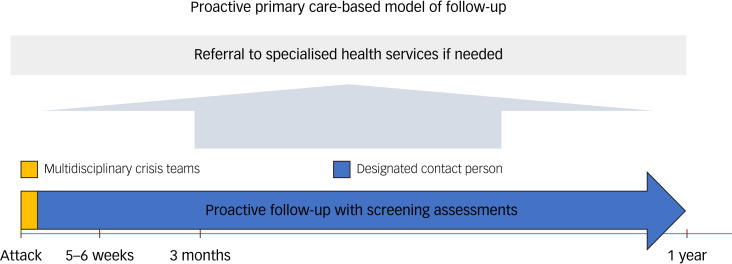
Illustration of the proactive primary care-based model of follow-up outlined in response to the Utøya attack. It was recommended that a municipal multidisciplinary crisis team contacted the survivors early after the attack. Next, that a designated contact person in their home municipality followed up the survivors throughout at least a year, including a minimum of three screening assessments at approximately 5–6 weeks, 3 months and 12 months after the attack. The intention was to use the lowest effective level of care, with referral to specialised health services if needed.

Our study population consisted of the survivors of the Utøya island attack. On 22 July 2011, two acts of terrorism were perpetrated in Norway. First, a bomb was detonated in the Oslo Government Quarter, killing eight individuals. Next, a shooting spree lasting more than an hour was committed on the small Utøya island at the summer camp of the youth Labour party, resulting in 69 fatalities, mostly of adolescents and young adults. The survivors were considered to be at high risk of developing post-attack health problems owing to several factors, including the high numbers of deaths and injuries, the long duration of the shooting, the young age of those affected, and the fact that they were designated targets of a terrorist, who was disguised as a police officer to mislead them into trusting him. Previous research has documented that the survivors were highly exposed to danger, and that the majority experienced loss of close ones in the attack.^21^ Additional factors of importance for the psychosocial follow-up included the survivors being geographically dispersed in rural and urban municipalities across the entire country and that many were about to move from their family to begin studies elsewhere a few weeks after the attack. To prevent or treat post-attack health problems and reduced functioning, the Norwegian Health Directorate recommended a proactive primary care-based outreach, where the survivors of the Utøya attack were to be immediately contacted by a multidisciplinary crisis team in their home municipality (Fig. 1).^9,18^ Moreover, it was advised that the survivors should be offered a designated contact person in their municipality. It was recommended that the contact person should contact the survivor as early as possible to secure continuity in the follow-up and observe the survivor's mental and physical health, social network and need for practical help. This proactive follow-up was recommended to last at least a year after the attack and to include a minimum of three screening assessments (at approximately 5–6 weeks, 3 months and 12 months post-attack). If considered necessary, the follow-up was recommended to continue beyond 1 year. The screening instrument contained questions on the survivor's experiences during the attack, sociodemographic information, access to social and practical support, and psychological and physical health, as well as functioning in the past 2 weeks. It focused on their experiences during the attack and did not cover other potentially traumatic events or stressors. It was developed using experiences from psychosocial care provision following Hurricane Katrina, school shootings and the 9/11 terrorist attacks.^9^ There was no standardised training of the contact persons, and the municipalities decided the professional background of the contact person. If the contact person did not have a clinical background, they were expected to ensure that health personnel conducted the screening assessments. The intention was to use the lowest effective level of care, with referral to specialised health services if needed. The proactive follow-up model was not mandatory, although it was recommended by the Health Directorate. Therefore, the municipalities were not obliged to implement it.

### Participants and procedures

**Fig. 2:**
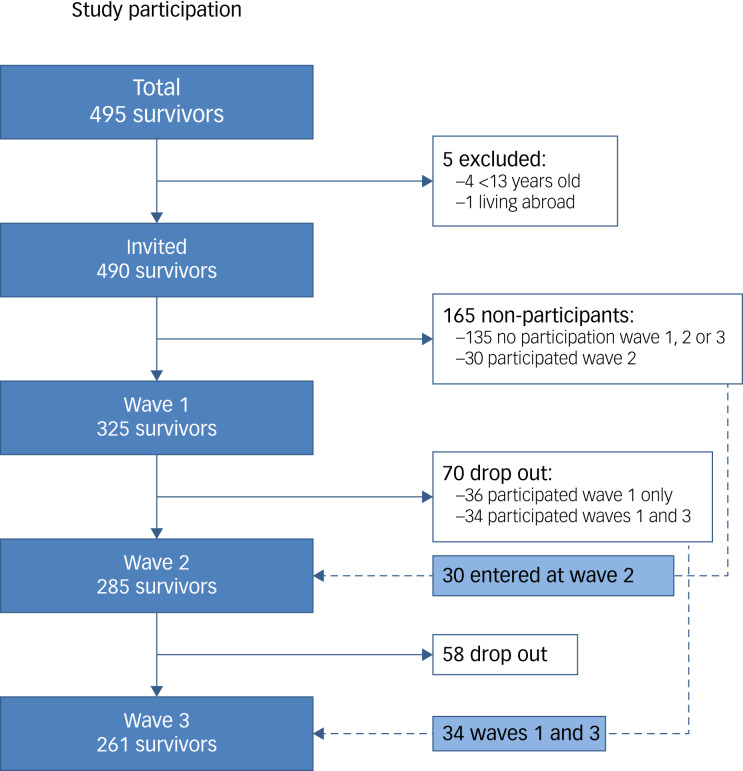
Flow chart of the study participation among survivors of the Utøya attack. Interviews were conducted at 4–5 months (wave 1), 14–15 months (wave 2) and 30–32 months (wave 3) after the attack. All the 490 eligible survivors were invited to participate in waves 1 and 2 (open cohort). Overall, 355 survivors (72%) participated in wave 1 or 2, and they were invited to participate in wave 3. Hence, no new participants joined the study in wave 3 (closed cohort).

Overall, 495 survivors who had been on Utøya island during the shooting were identified through police records. Invitations to participate in the study were sent by postal mail to 490 survivors; four aged <13 years and one living abroad were excluded. Our analyses included data from three waves of semi-structured interviews of the survivors conducted by trained personnel at 4–5 months (wave 1), 14–15 months (wave 2) and 31–32 months (wave 3) after the attacks. Furthermore, the interview data were linked with register-based data on consultations with specialised MHS from 3 years before to 3 years after the attack. All the 490 eligible survivors were invited to participate in waves 1 and 2, and 325 (66%) and 285 (58%) survivors participated, respectively. Overall, 355 survivors (72%) participated in wave 1 or 2, and they were all invited to participate in wave 3. Hence, no new participants joined the study in wave 3. Altogether, 261 (53%) of the survivors participated in wave 3; these comprised 207 who had also participated in both waves 1 and 2, 34 who had participated in wave 1 only, and 20 who had participated in wave 2 only. A flow chart of study participation is provided in Fig. 2. Register-based healthcare use data were collected at wave 3 for 255 survivors (52%). For six survivors, these data were not retrieved owing to lack of consent or absence of their personal identification number. The study participation and procedures have been described in detail previously.^8,22,23^

### Variables

Data on age, sex and place of residence were retrieved from police records. Age was measured at the time of the attack. Peripheral home municipality was defined as having a place of residence in a municipality with more than 45 min travelling time from a settlement with at least 15 000 inhabitants, in accordance with Statistics Norway's classification of centrality.^24^ Further, information on whether survivors had been admitted to hospital directly after the attack was verified by hospital records, and data on consultations with specialised MHS were obtained from the Norwegian Patient Registry (NPR). The other data were collected during the interviews.

### Interview data

#### Proactive follow-up

The participants reported whether they had had contact with a designated contact person at all survey waves. In the first wave, they reported whether they had had such contact since the attack (approximately the first 4–5 months after the attack). In wave 2, they were asked whether they had had such contact in the current year (2012), and in wave 3, they were asked whether they had had such contact during the past year (around 5–15 months and 20–32 months after the attack, respectively). The survivors’ experiences with their contact person were evaluated at wave 3 with questions on whether (a) they felt that the contact person treated them with care and consideration, (b) they had trust in the professional skills of the contact person, (c) they got enough time to talk and interact with the contact person, (d) it was easy to get in contact with the contact person, and (e) they felt that the contact with the contact person was useful. Satisfaction with the overall follow-up was assessed at wave 3 with the question: ‘Overall, were the help and treatment you received after the terrorist attack satisfactory?’. Response alternatives were ‘Not at all’, ‘To a small extent’, ‘To some extent’, ‘To a large extent’, and ‘To a very large extent’. Owing to the small numbers of respondents for some response alternatives, a dichotomous variable for satisfaction was applied, in which the latter two response alternatives were merged into one category and the remaining three into another.

#### Origin

Survivors with both parents born outside Norway were classified as having non-Norwegian origin, in accordance with Statistics Norway's definition.

#### Economy

The survivors’ financial situation was evaluated by a question on how they perceived their own (for survivors who did not live with parents) or their parents’ (for survivors who lived with parents) economic status compared with that of others. There were five response categories, which were dichotomised into financially disadvantaged yes (much or somewhat poorer) and no (similar, somewhat better or much better).

#### Exposure

Attack exposure was evaluated at wave 1 by a sum score of 13 potentially traumatic events occurring during the attack. Participants who entered the study in wave 2 answered questions on exposure then. The exposure score sum has been shown to be independently associated with mental health problems.^21^

#### Symptoms

Post-traumatic stress reactions in the preceding month were assessed by the University of California at Los Angeles Post-traumatic Stress Disorder Reaction Index.^25^ Its total score covers 17 items that conform to the DSM-IV symptoms of post-traumatic stress disorder. Each item is rated on a five-point Likert scale from 0 (never) to 4 (most of the time).^26^ Three of the items have two alternative phrasings; the item with the highest score is used to calculate the total score. This analysis covered the mean scores (Cronbach's alphas: waves 1 and 2 = 0.89 and wave 3 = 0.91).^8^ Symptoms of anxiety and depression were evaluated with the Hopkins Symptom Checklist-8 (SCL-8). SCL-8 is a short version of the SCL-25, covering symptoms of anxiety and depression in the past 2 weeks using eight items rated from 1 (not bothered) to 4 (very bothered).^27^ Cronbach's alphas for the mean scores were 0.85 (wave 1), 0.89 (wave 2) and 0.90 (wave 3).^8^ High psychometric qualities of the short versions of the SCL have been documented in population-based studies.^28^ Somatic symptoms in the past 2 weeks were evaluated by a short version of the Children's Somatic Symptoms Inventory.^29^ Its eight items covered pain in the stomach, head, lower back and arms and/or legs; faintness and/or dizziness; rapid heartbeat; nausea and/or stomach problems; and weakness. The items were rated on a scale from 1 (not bothered) to 4 (very bothered). Cronbach's alphas for the mean scores were 0.77 (wave 1), 0.78 (wave 2) and 0.77 (wave 3).^8^ Social support was measured using seven items from the Duke University of North Carolina Functional Social Support Questionnaire (FSSQ-7) scored from 1 (much less than I would like) to 5 (as much as I would like).^30^ Cronbach's alphas for the mean scores were 0.79 (wave 1), 0.77 (wave 2) and 0.80 (wave 3).^8^

### Register-based data

In this study, we linked the survivors’ interview data with register-based data from the NPR on consultations with specialised MHS from 3 years before until 3 years after the attack (22 July 2008–21 July 2014). All Norwegian residents have a personal identification number registered in encrypted form in the health registers. This enabled us to link the survivors’ interview data with their healthcare use data. NPR covers activity data from all specialist healthcare institutions in Norway, including records of all consultations and admissions in government-owned hospitals and out-patient clinics, as well as consultations with private contract specialists who receive government reimbursement. Reporting of information on individual patient care is mandatory and is linked to the governmental reimbursement system.^31,32^ We certified the records on consultations with private contract specialists in NPR with corresponding records from the Norwegian Health Economics Administration database to rectify a small percentage of missing data in NPR.^31,32^ We collected data on the survivors’ use of specialised child and adolescent and adult psychiatric services and merged them into one category: MHS.

### Ethics

Parental consent was required before survivors <16 years old could participate in the study. Survivors aged ≥16 years provided written informed consent before participating. The interviews were conducted by health practitioners who received designated training for this study at 1-day seminars ahead of the interview waves and were invited to attend meetings afterwards to share experiences. If unmet needs were discovered during the interviews, the interviewers were instructed to offer help to the survivors with contacting appropriate services. In addition, there was a telephone line for the interviewers to receive support if needed. The authors assert that all procedures contributing to this work comply with the ethical standards of the relevant national and institutional committees on human experimentation and with the Helsinki Declaration of 1975, as revised in 2013. All procedures involving human subjects were approved by the Regional Committees for Medical and Health Research Ethics Southeast and North in Norway (approval numbers REK 2011/1625 and REK 2014/246).

### Analysis

Characteristics of the survivors were compared by whether they had had contact with a designated contact person in the early, intermediate and long-term phases post-attack using Pearson's chi-squared tests for categorical variables and independent *t*-tests for continuous variables. To examine MHS use before and after each interview wave, we linked the participants’ dates of interviews with the register data. If a participant did not respond in all waves, the median date of interview of the sample was used as a proxy for estimation of MHS use in the corresponding time period. Percentages were calculated based on the total number of answers for each item. We used a two-sided statistical significance level of 0.05 and conducted the analyses using IBM SPSS version 29.0.0.0.

## Results

Characteristics of survivors according to whether or not they had contact with a designated contact person in the first 5 months, 5–15 months and 20–32 months after the attack are displayed in Tables 1A–C. Survivors who reported contact with a designated contact person during the first 5 months post-attack had lower levels of anxiety and/or depression symptoms at 14–15 months compared with those who did not have a designated contact person in the first 5 months (Table 1A). Still, long-term satisfaction with the overall follow-up did not differ by whether the survivors had a contact person in the first 5 months. By contrast, the survivors who had contact with a designated contact person at 5–15 months post-attack were more likely to report higher levels of anxiety and/or depression symptoms at 30–32 months post-attack, but higher satisfaction with the overall follow-up at long-term, compared with those who did not (Table 1B). There were no differences in sociodemographic factors according to whether the survivors had a contact person in the early or intermediary aftermath, i.e. during the period of recommended proactive follow-up. However, the 55 (22%) survivors who still reported contact with a designated contact person at 20–32 months post-attack were more likely to be financially disadvantaged (Table 1C). In all time periods, there were no statistically significant differences between the survivors who had or did not have a designated contact person according to exposure, post-traumatic stress symptoms, somatic symptoms, social support, hospital admission directly after the attack, or whether they lived in a peripheral (rural) municipality of residence. The latter two variables are not displayed in the tables owing to low counts (<5) in one of their categories.

**Table 1A:** Characteristics of survivors by whether they reported having had contact with a designated contact person at 0–5 months after the Utøya attack among the 314 (96.6%) participants in wave 1 who answered the question on contact person

	Contact in first 5 months (*n* = 314)		
		Yes (*n* = 263)	No (*n* = 51)	
Characteristics of survivors		*n* (%)	*n* (%)	*P*-value
Sex (*n* = 314)	Female	123 (46.8)	26 (51.0)	0.581
	Male	140 (53.2)	25 (49.0)	
<18 years old at the attack (*n* = 314)	Yes	127 (48.3)	20 (39.2)	0.235
	No	136 (51.7)	31 (60.8)	
Non-Norwegian origin (*n* = 310)	Yes	29 (11.1)	7 (14.6)	0.485
	No	233 (88.9)	41 (85.4)	
Financially disadvantaged (*n* = 315)	Yes	54 (21.0)	12 (25.0)	0.538
	No	203 (79.0)	36 (75.0)	
Relocation 0–5 months after attack (*n* = 311)	Yes	34 (13.0)	7 (14.0)	0.852
	No	227 (87.0)	43 (86.0)	
Overall satisfaction with help assessed at	To a high/very high extent	129 (65.8)	26 (70.3)	0.599
30–32 months (*n* = 233)	Not at all/to a little or some extent	67 (34.2)	11 (29.7)	
		Mean (s.d.)	Mean (s.d.)	
Mean sum of exposure (0–13) (*n* = 314)		8.44 (2.26)	8.59 (2.06)	0.666
Post-traumatic stress reaction (PTSD-RI)	Wave 1 (*n* = 314)	26.26 (12.40)	27.48 (11.51)	0.516
	Wave 2 (*n* = 248)	20.05 (11.32)	23.60 (11.03)	0.064
	Wave 3 (*n* = 234)	18.95 (12.30)	21.41 (11.11)	0.254
Anxiety/depression symptoms (SCL-8)	Wave 1 (*n* = 314)	2.06 (0.68)	2.13 (0.57)	0.454
	Wave 2 (*n* = 248)	1.74 (0.60)	1.97 (0.70)	0.029
	Wave 3 (*n* = 234)	1.69 (0.62)	1.89 (0.71)	0.081
Somatic symptoms (CSSI-8)	Wave 1 (*n* = 314)	1.72 (0.55)	1.70 (0.53)	0.756
	Wave 2 (*n* = 248)	1.60 (0.49)	1.71 (0.44)	0.192
	Wave 3 (*n* = 234)	1.50 (0.44)	1.65 (0.43)	0.055
Social support	Wave 1 (*n* = 314)	4.58 (0.56)	4.46 (0.63)	0.180
(FSSQ-7 mean score)	Wave 2 (*n* = 248)	4.58 (0.58)	4.50 (0.70)	0.466
	Wave 3 (*n* = 234)	4.54 (0.56)	4.41 (0.75)	0.232

PTSD-RI, Post-traumatic Stress Disorder Reaction Index; SCL-8, Hopkins Symptom Checklist-8; CSSI-8, Children's Somatic Symptoms Inventory; FSSQ-7, Functional Social Support Questionnaire.

**Table 1B:** Characteristics of survivors by whether they reported having had contact with a designated contact person at 5–15 months after the Utøya attack among the 275 (96.5%) participants in wave 2 who answered the question on contact person

	Contact in months 5–15 (*n* = 275)		
Characteristics of survivors		Yes (*n* = 152)	No (*n* = 123)	
		*n* (%)	*n* (%)	*P*-value
Sex (*n* = 275)	Female	69 (45.4)	61 (49.6)	0.488
	Male	83 (54.6)	62 (50.4)	
<18 years old at the attack (*n* = 275)	Yes	81 (53.3)	68 (55.3)	0.741
	No	71 (46.7)	55 (44.7)	
Non-Norwegian origin (*n* = 269)	Yes	12 (8.0)	13 (10.9)	0.412
	No	138 (92.0)	106 (89.1)	
Financially disadvantaged (*n* = 268)	Yes	33 (22.3)	22 (18.3)	0.424
	No	115 (77.7)	98 (81.7)	
Relocation 0–5 months after attack (*n* = 245)	Yes	16 (11.9)	17 (15.5)	0.411
	No	119 (88.1)	93 (84.5)	
Overall satisfaction with help assessed at	To a high/very high extent	95 (75.4)	54 (57.4)	0.005
30–32 months (*n* = 220)	Not at all/to a little or some extent	31 (24.6)	40 (42.6)	
		Mean (s.d.)	Mean (s.d.)	
Mean sum of exposure (0–13) (*n* = 279)		8.33 (2.29)	8.36 (2.20)	0.901
Post-traumatic stress reactions (PTSD-RI)	Wave 1 (*n* = 247)	26.11 (12.54)	26.05 (11.64)	0.969
	Wave 2 (*n* = 275)	21.14 (11.74)	21.41 (11.45)	0.848
	Wave 3 (*n* = 221)	19.83 (13.20)	18.24 (10.52)	0.321
Anxiety/depression symptoms (SCL-8)	Wave 1 (*n* = 247)	2.06 (0.67)	2.02 (0.62)	0.608
	Wave 2 (*n* = 275)	1.78 (0.66)	1.82 (0.61)	0.608
	Wave 3 (*n* = 221)	1.80 (0.67)	1.60 (0.56)	0.016
Somatic symptoms (CSSI-8)	Wave 1 (*n* = 247)	1.75 (0.58)	1.69 (0.51)	0.403
	Wave 2 (*n* = 275)	1.66 (0.56)	1.63 (0.45)	0.643
	Wave 3 (*n* = 221)	1.59 (0.54)	1.49 (0.39)	0.133
Social support	Wave 1 (*n* = 247)	4.55 (0.58)	4.57 (0.54)	0.764
(FSSQ-7 mean score)	Wave 2 (*n* = 275)	4.57 (0.58)	4.54 (0.62)	0.732
	Wave 3 (*n* = 221)	4.52 (0.58)	4.59 (0.52)	0.345

PTSD-RI, Post-traumatic Stress Disorder Reaction Index; SCL-8, Hopkins Symptom Checklist-8; CSSI-8, Children's Somatic Symptoms Inventory; FSSQ-7, Functional Social Support Questionnaire.

**Table 1C:** Characteristics of survivors by whether they reported having had contact with a designated contact person at 20–32 months after the Utøya attack among the 254 (97.3%) participants in wave 3 who answered the question on contact person

	Contact in months 20–32 (*n* = 254)		
Characteristics of survivors		Yes (*n* = 55)	No (*n* = 199)	
		*n* (%)	*n (%)*	*P*-value
Sex (*n* = 254)	Female	28 (50.9)	93 (46.7)	0.583
	Male	27 (49.1)	106 (53.3)	
<18 years old at the attack (*n* = 254)	Yes	25 (45.5)	87 (43.7)	0.818
	No	30 (54.5)	112 (56.3)	
Non-Norwegian origin (*n* = 251)	Yes	7 (13.0)	14 (7.1)	0.169
	No	47 (87.0)	183 (92.9)	
Financially disadvantaged (*n* = 248)	Yes	16 (30.2)	33 (16.9)	0.031
	No	37 (69.8)	162 (83.1)	
Relocation 0–5 months after attack (*n* = 233)	Yes	8 (15.1)	21 (11.7)	0.506
	No	45 (84.9)	159 (88.3)	
Overall satisfaction with help assessed at	To a high/very high extent	43 (78.2)	128 (64.6)	0.058
30–32 months (*n* = 253)	Not at all/to a little or some extent	12 (21.8)	70 (35.4)	
		Mean (s.d.)	Mean (s.d.)	
Mean sum of exposure (0–13) (*n* = 251)		9.00 (2.31)	8.35 (2.10)	0.051
Post-traumatic stress reactions (PTSD-RI)	Wave 1 (*n* = 234)	26.91 (13.10)	25.94 (11.70)	0.624
	Wave 2 (*n* = 220)	22.09 (11.78)	20.67 (11.62)	0.469
	Wave 3 (*n* = 254)	20.63 (12.85)	18.94 (12.17)	0.369
Anxiety/depression symptoms (SCL-8)	Wave 1 (*n* = 234)	2.08 (0.66)	2.06 (0.63)	0.839
	Wave 2 (*n* = 220)	1.84 (0.65)	1.81 (0.65)	0.717
	Wave 3 (*n* = 254)	1.80 (0.65)	1.70 (0.65)	0.305
Somatic symptoms (CSSI-8)	Wave 1 (*n* = 234)	1.84 (0.57)	1.69 (0.54)	0.064
	Wave 2 (*n* = 220)	1.77 (0.50)	1.62 (0.52)	0.079
	Wave 3 (*n* = 254)	1.58 (0.47)	1.53 (0.47)	0.467
Social support	Wave 1 (*n* = 234)	4.59 (0.55)	4.56 (0.54)	0.736
(FSSQ-7 mean score)	Wave 2 (*n* = 220)	4.60 (0.54)	4.59 (0.51)	0.892
	Wave 3 (*n* = 254)	4.49 (0.58)	4.54 (0.58)	0.635

PTSD-RI, Post-traumatic Stress Disorder Reaction Index; SCL-8, Hopkins Symptom Checklist-8; CSSI-8, Children's Somatic Symptoms Inventory; FSSQ-7, Functional Social Support Questionnaire.

**Fig. 3:**
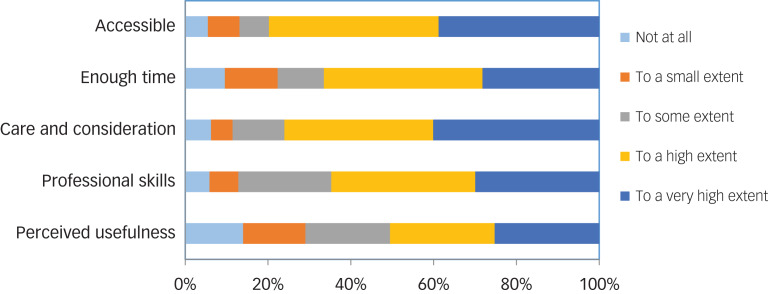
The survivors’ overall experiences with the designated contact person reported at wave 3 (30–32 months after the attack).

Furthermore, we found no statistical differences by having had a contact person and participation or non-participation in any of the three interview waves among the overall 355 survivors who participated in at least one wave. Among the 202 (79%) survivors who in wave 3 reported that they had had a designated contact person post-attack, 13 (6%) declared that their contact person was a general practitioner (GP), 12 (6%) a school nurse or someone from the school health services, 91 (45%) other municipal personnel, 29 (14%) a psychiatrist, psychologist or psychiatric nurse in the specialised MHS, and 13 (6%) another professional; 41 (20%) reported that they did not know their contact person's professional background, and three did not respond. Table 2 presents register-based data on MHS consultations by contact with a designated contact person in different time periods. Survivors who reported contact with a contact person 0–5 months post-attack were significantly less likely to have consulted MHS in the same period. In the other time periods, there were no significant differences in MHS consultations by whether or not the survivors had a contact person. After excluding the survivors who reported that their contact person had been from the MHS, there were no significant differences in MHS use associated with having had a contact person in any time period. Figure 3 illustrates the survivors’ overall experiences with their contact person among those who at wave 3 reported having had a designated contact person. Around 80% reported that their contact person was accessible to a high or very high extent, and 76% that the contact person showed care and consideration to a high or very high extent, whereas 66% reported that they received enough time with their contact person to a high or very high extent, and 65% that their contact person had adequate professional skills to a high or very high extent. Approximately half reported that it had been useful to a high or very high extent to have had a contact person, whereas almost one-third reported no or little usefulness. Table 3 displays the exact number of respondents for each category shown in Fig. 3.

**Table 2:** Consultations (≥1) with the specialised mental health services (MHS) according to whether survivors of the Utøya attack had contact with a designated contact person in the first 5 months, between 5 and 15 months, and between 20 and 32 months after the attack. Information on MHS consultations was drawn from register-based data

	Contact in first 5 months (*n* = 229)			Contact in months 5–15 (*n* = 217)			Contact in months 20–32 (*n* = 248)		
MHS consultations	Yes (*n* = 191)	No (*n* = 38)		Yes (*n* = 122)	No (*n* = 95)		Yes (*n* = 54)	No (*n* = 194)	
	*n* (%)	*n* (%)	*P*-value	*n* (%)	*n* (%)	*P-*value	*n* (%)	*n* (%)	*P-*value
Pre-attack	Yes	25 (13)	5 (13)	0.991	20 (16)	12 (13)	0.438	12 (22)	26 (26)	0.112
(preceding 3 years)	No	166 (87)	33 (87)		102 (84)	83 (87)		42 (78)	168 (74)	
Between attack and wave 1	Yes	101 (53)	27 (71)	0.039	66 (54)	56 (59)	0.475	30 (56)	111 (57)	0.827
(0–5 months post-attack)	No	90 (47)	11 (29)		56 (46)	39 (41)		24 (44)	83 (43)	
Between waves 1 and 2	Yes	106 (55.5)	27 (71)	0.076	76 (62)	52 (55)	0.261	36 (67)	112 (58)	0.236
(5–15 months post-attack)	No	85 (44.5)	11 (29)		46 (38)	43 (45)		18 (33)	82 (42)	
Between waves 2 and 3	Yes	82 (43.5)	19 (50)	0.459	64 (52.5)	39 (41)	0.095	29 (54)	86 (56)	0.222
(15–32 months post-attack)	No	108 (56.5)	19 (50)		58 (47.5)	56 (59)		25 (46)	108 (44)	
Between attack and wave 3	Yes	132 (64)	30 (79)	0.223	88 (72)	65 (68)	0.552	40 (74)	139 (72)	0.725
(0–32 months after attack)	No	59 (31)	8 (21)		43 (28)	30 (32)		14 (26)	55 (28)

**Table 3:** Number of respondents for each category in Fig. 3 regarding the survivors’ perceived experiences with their contact person

	Not at all	To a small extent	To some extent	To a high extent	To a very high extent	Total
Perceived usefulness	26	28	38	47	47	186
Professional skills	11	13	42	65	56	187
Care and consideration	12	10	24	69	77	192
Enough time	18	24	21	72	53	188
Accessible	10	14	13	75	71	183

## Discussion

This study indicates that the proactive follow-up reached survivors of the Utøya attack across sociodemographic characteristics during the recommended first year of follow-up. Nevertheless, there were some differences between the survivors who received proactive follow-up and those who did not, and the factors associated with having a contact person varied across different post-attack periods. In the first 4–5 months, 84% of the survivors had contact with a contact person. Those who did not were more likely to receive specialised MHS, and to have higher levels of anxiety and/or depression symptoms in the ensuing months than survivors who had a contact person. In the following period, until around 15 months post-attack, 55% of the survivors reported having had a contact person. Those who did not were more likely to be less satisfied with the overall help they received post-attack but were also less likely to have anxiety and/or depression symptoms in the long-term than those who had a contact person. At long-term follow-up (20–32 months post-attack), 22% of the survivors still had contact with a designated contact person. Those who did were more likely to be financially disadvantaged. Among survivors who reported having had a contact person after the attack, around half reported that this had been highly or very highly useful, whereas nearly one-third reported that it was of little use or not at all useful.

### Interpretation and comparison

In line with prior research, this study indicates that the need for follow-up of survivors of severe, potentially traumatic events may last for several years.^6,32,33^ Although the minimum recommended period for proactive follow-up was 1 year, 22% of the survivors still had contact with their contact person 20–32 months post-attack. This suggests that many municipalities arranged for follow-up beyond the first year. A recent register-based study reported that the survivors of the Utøya attack also had markedly increased consultation rates with MHS and GPs for several years post-attack.^32^ In contrast to factors previously found to be associated with healthcare use for common mental disorders,^32,34^ we found no significant differences by sociodemographic factors according to whether the survivors had a contact person in the first 15 months. Still, nearly half of the survivors were not followed up by a contact person throughout the first year post-attack, although this was recommended in the proactive outreach model. We did not have information about the causes for this, and there may have been several explanations. On the one hand, some survivors may have declined to be recontacted in the long term. As nearly one-third of the survivors with a contact person perceived this contact as of little use or not at all useful, they may have preferred to end the follow-up. On the other hand, some municipalities may not have offered proactive follow-up throughout the first year. A qualitative analysis demonstrated that the survivors had contrasting experiences with their contact person.^35^ Among the positive aspects, it was described as useful to have one designated person to take responsibility and coordinate the care post-attack. Furthermore, the contact person often helped with practical issues, for instance, organising support at school or work and contacting different types of health service, such as the GP and MHS. Still, some survivors mentioned that the follow-up was not sufficiently proactive and that they would have preferred a more frequent follow-up lasting beyond the first year post-attack. In certain cases, the contact person only contacted the survivor once, or sent a letter with contact information, leaving it up to the survivor to take contact. Other survivors reported that they declined help in the early phase but felt the need later, when it could be more difficult to access help. This indicates that improvements are required in the long-term proactive offer of help. Whereas some survivors expressed that they did not receive enough help, others found that there was too much contact. Indeed, the quantity and quality of the follow-up from different contact persons seemed to vary considerably. A former quantitative analysis found that one in five (20%) survivors scored their healthcare needs for attack-related psychological concerns or problems as greater than the help they had received, whereas more than one in three (35%) scored their needs as lower than the help they had received.^8^ Concerning attack-related physical health problems, 14% of the survivors rated their healthcare needs as greater than and 16% as less than the help they had received.^8^ Hence, a substantial proportion of survivors perceived their healthcare needs as greater than the help they received, and a slightly higher proportion perceived their healthcare needs as lower than the help they received. This suggests that the healthcare provided after terrorist attacks and disasters could be better targeted and tailored.

Satisfaction with overall follow-up did not significantly differ by whether the survivors had a contact person 0–5 months post-attack, but survivors who had a contact person 5–15 months post-attack were more satisfied with the overall follow-up in the long term compared with those who did not. The survivors who did not have a contact person early after the attack were, however, more likely to receive MHS consultations during the same period. This might explain why they were equally satisfied with the overall follow-up compared with survivors who had a contact person. Regarding the relationship between having a contact person and symptoms of anxiety and/or depression, the findings varied across different time periods. Survivors who had a contact person in the early aftermath were less likely to report symptoms of anxiety or depression at around 14–15 months post-attack (wave 2). Conversely, in the subsequent period, those who had a contact person at 5–15 months post-attack were more likely to report symptoms of anxiety or depression at approximately 30–32 months post-attack (wave 3). Owing to conflicting results, no conclusion could be reached regarding whether having a contact person had any impact on the survivors’ health. The higher long-term satisfaction with the overall follow-up among survivors who had a contact person in the intermediary period post-attack could indicate that providing a contact person for survivors over time increases satisfaction with follow-up. Nonetheless, it is also possible that some of the survivors who did not have a contact person after the early phase themselves declined further follow-up owing to not being satisfied with this follow-up. Financially disadvantaged survivors were more likely to have a contact person beyond the recommended first year of follow-up. We lack information on the reasons for this occurrence, or on whether this extended follow-up from the contact person was offered proactively or in response to a request for additional support. Nonetheless, we interpret this finding as indicating that easily accessible, free-of-charge psychosocial support in the long-term is especially important for financially disadvantaged survivors, even in a country with universal health coverage, such as Norway.

The follow-up model outlined in response to the Utøya attack differed from certain recommendations in international guidelines for post-disaster psychosocial care. For instance, the European Network of Traumatic Stress guidelines for psychosocial care following disaster and major incidents developed in 2008 state that there should not be formal screening of everyone affected, but that helpers should be aware of the importance of identifying individuals with significant difficulties.^10^ By contrast, it was recommended that everyone who had been on Utøya during the shooting should be followed up proactively with screenings throughout a minimum of 1 year, irrespective of their symptom levels and functioning. The more comprehensive follow-up of the survivors of the Utøya attack may have been due to the severity of the incident and the young age of those concerned. Nevertheless, the fact that approximately one-third reported that the follow-up from the contact person was of little use or not at all useful to them could indicate that not all those exposed to such a severe event would benefit from proactive follow-up. A challenge with proactive outreach is to decide who should receive proactive follow-up and screening assessments and who should not. The recently updated BMJ Best Practice guidance on mental health response to disasters and other critical incidents suggests proactive follow-up based on screening assessments of disaster survivors in the immediate days post-disaster.^36^ Next, survivors deemed to be at risk based on the initial screening should receive follow-up phone screening after approximately 4 weeks. Finally, at-risk survivors who have high symptom levels should be scheduled in-person treatment appointments. Notwithstanding, as recognised in the guidelines, there is still a major need for methodologically sound scientific evidence regarding what would constitute the best practices for post-disaster psychosocial follow-up.

Another divergence from international guidelines was that there was no specific mention of the training required to serve as a contact person in the proactive follow-up model proposed after the Utøya attack. As such, it is possible that a lack of tailored training contributed to the disparities in the help and support provided by the contact persons.

Finally, the Norwegian Health Directorate recommended to the municipalities that they should implement a proactive outreach model with a designated contact person for all survivors, but this was not mandatory.^9^ This may have contributed to geographical differences in the healthcare offer, as some survivors may not have been offered a contact person simply owing to their municipality of residence.^37^ The fact that a specific model of follow-up was recommended by the Health Directorate may have created an expectation among survivors of receiving a contact person, which again may have contributed to lower satisfaction among survivors who were not offered a contact person.

### Strengths and limitations

The strengths of this study included its combination of self-reported and register-based data. It covered three waves of interviews linked with pre- and post-attack register-based data on specialised MHS use in a relatively large group of young survivors who had been exposed to the same potentially traumatic event. The longitudinal design enabled us to study changes over time. Furthermore, the inclusion of register-based data yielded accurate information on MHS use that was not prone to recall bias. However, the information on the contact persons was based on self-reports and could thus have been affected by recall bias. Another limitation of the study was that it did not include all survivors, and so there may have been selection bias. Prior research suggests that there were no statistically significant differences between participants and non-participants by age, gender, peripheral versus central residence, or hospital admission directly after the attack.^22^ Survivors who had participated in wave 1 or 2 but not in wave 3 were significantly more likely to be of non-Norwegian origin. However, they did not differ regarding symptom levels. We could not determine whether the higher attrition among survivors of non-Norwegian origin resulted in selection bias, but the validity of the results may be more uncertain for participants of non-Norwegian origin. Lower response rates among immigrant survivors have previously been found in a longitudinal study of survivors of a fireworks disaster in The Netherlands.^38^ Similarly, response rates have been found to be lower among immigrants compared with non-immigrants in population-based research.^39^ It is uncertain why attrition was higher among immigrant survivors. Factors that have been suggested as possible explanations for lower response rates among immigrants include language skills, lower perceived ability to answer surveys, lower perceived ability to influence policy, and distrust in public institutions.^40^ We do not know whether study participants were more or less likely to have had a contact person than non-participants. Study participation may, however, have increased the likelihood of referrals to healthcare if unmet needs were discovered during the interviews. Furthermore, we could not assess the content of the proactive follow-up. Thus, there may have been considerable variation in what the contact with the contact person covered regarding the questions used in our analyses. Our assessment of the use of contact persons was based on self-reported data from the survivors; we did not have access to data on the actual implementation of screening assessments. Furthermore, we did not have information on whether survivors without a designated contact person might have received screening assessments through self-referral to public or private health services, for instance. Finally, external validity may depend on the health system and cultural characteristics.

### Clinical implications and future research

Proactive outreach, in contrast to self-referral, allowed survivors across sociodemographic characteristics to be reached during the recommended first year of follow-up. This could have the benefit of meeting health needs in individuals and population groups less inclined to seek healthcare. Our findings further emphasise the importance of monitoring whether recommendations for psychosocial care are indeed implemented and ensuring that all survivors are offered follow-up. Particular attention is warranted concerning long-term follow-up and the need to facilitate access to help if problems surface in the long term. There appeared to be considerable variation in the quantity, quality and duration of follow-up from the contact persons. This underscores the importance of implementing tailored training for all providers of psychosocial care and proactive outreach, as well as providing sufficient information on what the follow-up should contain and support with its implementation.

The current study, in line with most prior research on post-disaster psychosocial support, could not reach a conclusion regarding the potential health and social benefits or disadvantages of the interventions implemented. Therefore, we do not have sufficient information to either recommend or discourage this proactive primary-care-based model of follow-up from designated municipal contact persons. To strengthen future public health preparedness to disasters, it is essential to plan how to implement proper monitoring, evaluation and research that could help guide the provision of post-disaster psychosocial care. Finally, in future research, it will be important to collect data from providers of psychosocial care to gain knowledge about their experiences and challenges with respect to the provision of care.

## Data Availability

The data are not publicly available owing to restrictions related to their containing information that could compromise the privacy of research participants.
